# A Flexible Double-Sided Curvature Sensor Array for Use in Soft Robotics

**DOI:** 10.3390/s24113475

**Published:** 2024-05-28

**Authors:** Racha Benarrait, Muneeb Ullah-Khan, Jérémy Terrien, Hani Al Hajjar, Frédéric Lamarque, Andreas Dietzel

**Affiliations:** 1 Institute of Microtechnology, Technische Universität Braunschweig, 38124 Braunschweig, Germany; a.dietzel@tu-braunschweig.de; 2 Université de Technologie de Compiègne, CNRS FRE 2012, Roberval (Mechanics Energy and Electricity), Centre de Recherche Royallieu, CS 60319, 60203 Compiègne Cedex, France; muneeb-ullah.khan@utc.fr (M.U.-K.); jeremy.terrien@utc.fr (J.T.); hani.al-hajjar@utc.fr (H.A.H.); frederic.lamarque@utc.fr (F.L.)

**Keywords:** piezoresistive sensor, micro-electro-mechanical systems, microfabrication, flexible electronics, flexible sensor, soft robotics, shape memory alloy, strain gauge

## Abstract

This paper describes the design, fabrication, integration, characterization, and demonstration of a novel flexible double-sided curvature sensor array for use in soft robotics. The paper explores the performance and potential applications of a piezoresistive sensor array consisting of four gold strain gauges on a flexible polyimide (PI) substrate arranged in a Wheatstone bridge configuration. Multiple sensor strips were arranged like the fingers of a hand. Integrating Shape Memory Alloy (SMA) foils alongside the fingers was explored to mimic a human hand-gripping motion controlled with temperature, while curvature sensor array strips measure the resulting finger shapes. Moreover, object sensing in a flexible granular material gripper was demonstrated. The sensors were embedded within Polydimethylsiloxane (PDMS) to enhance their tactile feel and adhesive properties. The findings of this study are promising for future applications, particularly in robotics and prosthetics, as the ability to accurately mimic human hand movements and reconstruct sensor surfaces paves the way for robotic hand functionality.

## 1. Introduction

Soft robotic grippers mimic the dexterity of human hands and can handle small, soft, and irregularly shaped objects that rigid grippers cannot [1]. To improve their performance, integrated sensors must provide specific information about their adapting shape and their movements. Sensors using flexible and wearable electronics enable real-time and precise measurements as these sensor arrays can contribute to shape sensing and monitoring, gesture recognition, pressure mapping, object interaction, and feedback, enabling them to adjust their grip based on the shape or pressure of an object [2]. Piezoresistive sensors are a promising solution that can improve the overall performance of robotic grasping, enhancing their application in different fields such as industrial automation, healthcare, and assistive robotics [3].

In robotics, electronic skin, or e-skin, enhances the tactile perception of robotic systems, while touch-sensitive devices with superior flexibility can be developed in human–computer interaction. Electronic skin, or e-skin, is a flexible and stretchable electronic device miming human skin’s sensing capabilities. It detects stimuli like temperature, pressure, and touch, enabling applications in healthcare, robotics, and wearable technology [4,5,6,7].

The main challenge in designing e-skin is to create electrical sensors that can conform to the natural movements of human skin while maintaining electrical function. For this, researchers have explored various materials, from traditional metals to advanced conductive polymers and nanomaterials [8].

Conductive polymers are particularly notable for their exceptional stretchability. However, in order to enhance the electrical performance of these nanomaterials, their design strategies mainly focus on engineering the conductor structure and composition, including optimization of the arrangement of conductive elements and stretch-compliant features like serpentine patterns or microstructures. Serpentine structures are widely used in stretchable electronics to enhance the stretchability of rigid materials like conductive polymers. Thanks to their geometric design of interconnected loops and curves that maintain electrical conductivity, these structures can accommodate mechanical strain and deformation without compromising the material’s electronic properties [9].

Soft robots, often designed to mimic nature, are currently being developed for various applications. They leverage their flexibility and deformability to mimic the movements and functions of living organisms. However, their soft structure makes them prone to different types of damage, such as sharp objects, interfacial debonding, and fatigue, which limit their lifespan [10]. To address this issue, monitoring the movements of soft robots is a promising solution [11]. Soft robots can bend, stretch, and twist around obstacles, making them inherently safer but more challenging to control due to their multiple degrees of freedom. Soft robots can be monitored continuously to detect collisions or other irregularities, thus adjusting control parameters. This information can be used to improve the robots’ reliability, performance, and safety and inform iterative design improvements for more robust and durable soft robotic systems in the future. To achieve accurate control, soft robots must integrate sensors that detect their precise shape and state at any time. This will allow them to grasp unknown objects without requiring precise actuator positioning. However, creating sensors for soft robots is still challenging, as the sensors need to be soft and have limited influence on the mechanical properties of the actuator. One solution is to use flexible sensors, which can be embedded in soft robots to detect deformation and damage. However, these sensors are also susceptible to damage, just like the actuator in which they are embedded.

Soft robotics sensing is still underdeveloped compared to actuation, but integrating sensing and actuation directly into soft mechanisms can extend their applications and enable more complex robot behavior [12]. This integration should be performed without modifying the properties of the soft device and should be durable enough to last over many motion cycles [13].

Many different types of soft actuators using smart materials have been developed with a variety of capabilities and methods of fabrication. However, Shape Memory Alloys (SMAs) are unique due to their quiet operation and high-power density. Despite these advantages, they have been used rarely in soft robotics applications due to challenges such as activation temperature, complex control and integration, and limited speed of response. Nitinol, a popular SMA material, usually has a stroke of approximately 4–8% of its original length [14,15]. However, it is possible to convert this minor deformation into stronger out-of-plane deformations by integrating it into a polymeric matrix [16]. The design and fabrication of an integrated sensor system capable of discriminating among different stimuli for motion monitoring are challenging.

In this article, we present a novel flexible system that can measure local bending strains and has an integrated actuation using SMA foils. It can thereby mimic deformation and validate the sensors’ shape recognition functionality. This functionality is very important for soft robotics because the end-effector can deform when it comes into contact with objects to be manipulated. We first describe the investigations of the performance and potential applications of a piezoresistive sensor array consisting of four gold strain gauges arranged in a Wheatstone bridge configuration on a flexible polyimide (PI) substrate. The sensor array can measure the curvature of objects by providing voltage outputs, which are inversely proportional to the locally applied bending. A printed circuit board (PCB) has been designed to include an amplification circuit and offset compensation. The setup involves using data acquisition (DAQ) equipment, specifically NI LabVIEW, to collect output voltages from a single sensor strip at various curvatures. A separate setup has been established to characterize multiple sensor strips arranged like the fingers of a hand, including a microcontroller interfaced with the sensor strips. A MATLAB program interacts with the microcontroller and collects data from 20 sensor nodes simultaneously. A five-finger shaped printed circuit board has been designed to enhance connectivity and efficiency.

## 2. Materials and Methods

### 2.1. Piezoresistive Sensor Concept and Design

**Figure 1:**
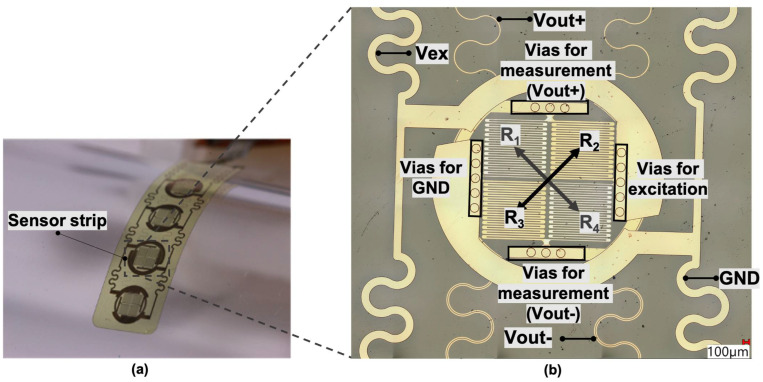
(**a**) Image of a longitudinal bending over a glass tube with 10 mm as radius. (**b**) An enlarged image of one single sensor node of the implemented full Wheatstone bridge.

The flexible sensor array utilizes the piezoresistive effect in meandering metallic conductors. The sensor strip consists of four nodes, and each node is composed of four resistors in a Wheatstone bridge configuration. The resistors R_2_ and R_3_ on the top surface and R_1_ and R_4_ on the bottom are placed diagonally, as shown in Figure 1. This concept with strain gauges placed on both sides of the neutral axis offers increased sensitivity compared to single-sided sensor arrays; moreover, thermal drifts can be compensated. While one resistor group experiences tensile strain, the other experiences compressive strain. The current through the bridge is balanced when the ratio of the resistance of two of the resistors is equal to the ratio of the resistance of the other two resistors. In an unbalanced situation a non-zero output voltage $V_{\mathrm{out}}$ can be measured as:

(1) $$V_{out}=\left(\frac{R_{4}}{R_{2}+R_{4}}-\frac{R_{3}}{R_{1}+R_{3}}\right)V_{ex}$$

where $V_{ex}$ is the excitation voltage applied to the bridge.

**Figure 2:**
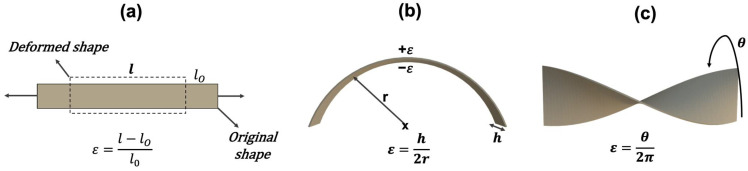
Strain induced by (**a**) foil stretching, (**b**) foil bending as for curvature sensing and (**c**) torsion.

This strain sensor can be utilized in various designs to sense stretching, bending, and twisting (see Figure 2). When bending the bridge with radius r, the output voltage of the bridge $V_{\mathrm{out}}$ can be measured as [17]:

(2) $$V_{out}\left(r\right)=A\cdot GF\cdot \frac{h}{2r}\cdot V_{ex}$$

where, in our case, $V_{\mathrm{ex}}$ = 2.5 V was the input voltage of the Wheatstone bridge, h = 11.5 µm the thickness of the layer between the sensors (R_2_ and R_3_ on top, R_1_ and R_4_ on bottom) and GF≈2 the gauge factor of the thin metal film conductor. $V_{\mathrm{out}}$ was amplified by the measurement electronics by a factor of A = 100.

### 2.2. Sensor Strip Fabrication Method

**Figure 3:**
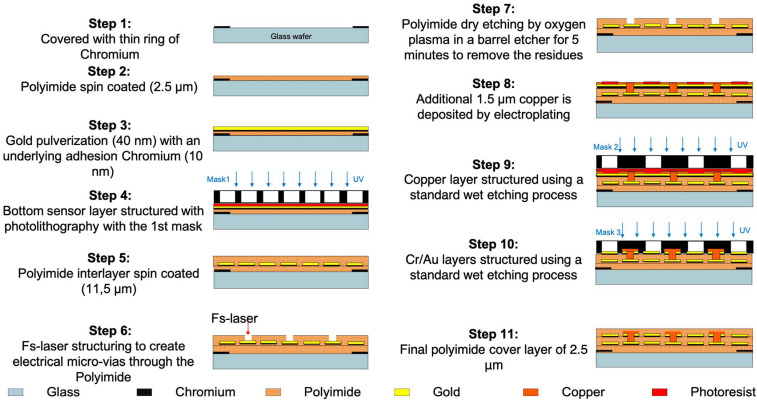
Microfabrication process of the sensor strips [18].

The process of creating the sensor strip is illustrated in Figure 3. The strip with four sensor nodes was developed on polyimide PI2610 (from HD MicroSystems, Neu-Isenburg, Germany) as the base material, which was processed as a liquid precursor on a rigid glass substrate. The sensitive elements were built up from sputter-deposited chromium (10 nm, for good adhesion) and gold (40 nm). The Cr/Au layers were structured using the photolithographic process followed by wet etching of the metal (an alkaline solution and an iodine solution are used for chromium and gold wet etching, respectively). The second sensor layer was realized similarly to the first sensor layer. To establish the bridge wiring, a femtosecond laser workstation 175 microSTRUCT C equipped with Light Conversion, Pharos-15W laser (from 3DMicromac, Chemnitz, Germany) was used to create electrical micro-vias through the polyimide. The vias were cleansed of residues by employing a barrel etcher (specifically, the 308 PC Barrel Etching System manufactured by STS Surface Technology Systems GmbH, Hückelhoven, Germany), which utilized a gas blend comprising CO_2_ and CF_4_. Electrical interconnections between the two upper and lower sensor layers were established using a copper layer measuring 1.5 μm in thickness and added through chemical electroplating, employing a current density of 15 mA/cm^2^ over 6 min. Finally, a 2.5 μm polyimide cover layer was applied before using a femtosecond laser for contour cutting of the thin flexible sensor foil. Removing it from the rigid glass wafer was then straightforward [18].

### 2.3. Sensor Evaluation Circuitry

**Figure 4:**
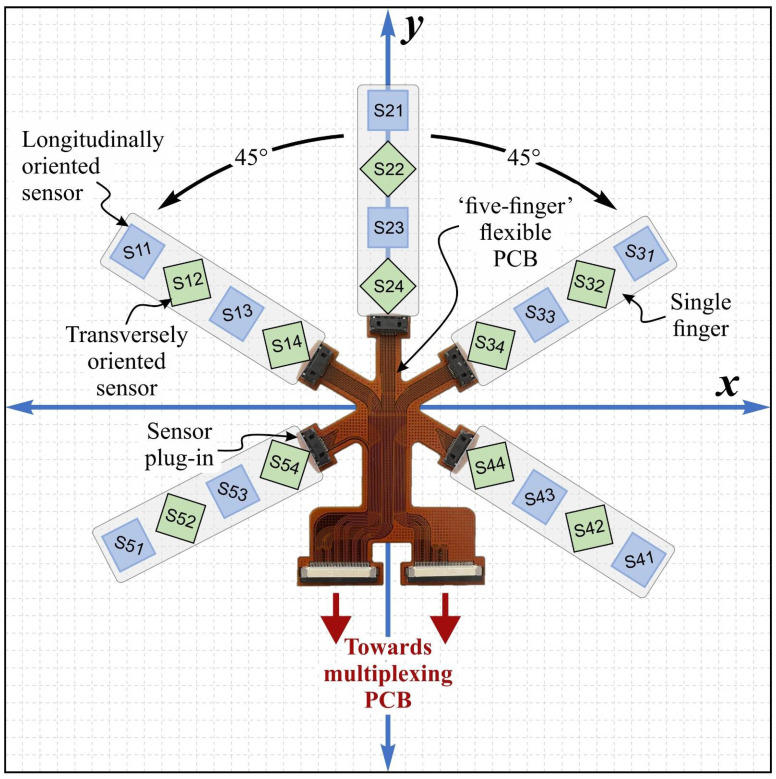
Photo of the flexible PCB with the sketched arrangement of the sensor nodes S11 to S44 using four sensor strips. Longitudinally oriented are the sensor nodes Sn1 and Sn3 (n = 1, …, 5), transversally orientated are sensor nodes Sn2 and Sn4 (n = 1, …, 5).

In order to achieve direct contact with varying parts shapes, a flexible PCB was designed. We chose a five-finger configuration-based flexible PCB design to monitor multiple flexible sensors (see Figure 4). Each sensor had 10 contacts, including eight contacts for the four Wheatstone bridge structures and two contacts for the power supply, making a total of 20 Wheatstone bridge structures and 50 connections for the five flexible sensors. The 50 contacts were connected to the flexible PCB by five FH63S-10S-0.5SH connectors from Mouser Electronics, Munich, Germany, and the signals were output to the next-level processor by two SD-54132-064 connectors from Digi-Key Electronics, München, Germany.

**Figure 5:**
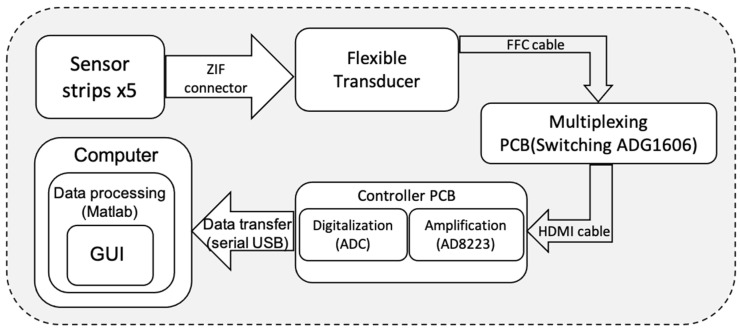
Schematic layout of the evaluation electronics.

Raw $V_{out}$ signals could be selected using the ADG1606 multiplexer. An AD8223 amplifier did magnify the weak signals. This amplified signal was digitized by an analog-to-digital converter (ADC). The system employed a serial USB interface to transfer the sensor data to a computer where MATLAB version R2020b could process the data and provide visualization (see Figure 5).

The data from five sensor strips each with four sensor nodes (arranged as sketched in Figure 4) were displayed in the form of a 3D graph. As Figure 4 shows, Sn1 and Sn3 (n = 1, …, 5) sensor nodes were longitudinally oriented, while Sn2 and Sn4 (n = 1, …, 5) were transversely oriented. A continuous surface shape was fitted to reconstruct the 3D shape of an object that was in contact with the measuring strips.

### 2.4. Shape Memory Alloy Actuated Robotic Fingers

**Figure 6:**
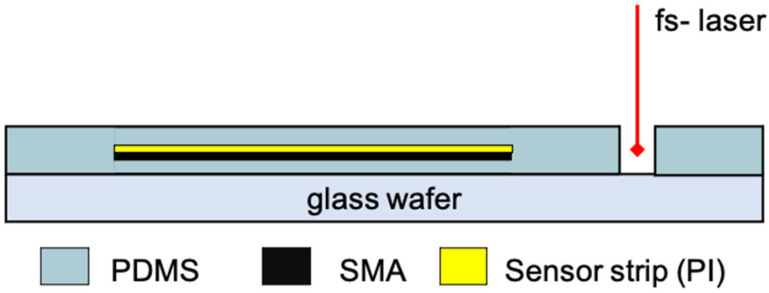
Schematic illustration of the sensor strip and SMA actuation strip embedding in polydimethylsiloxane (PDMS).

Nickel titanium alloy foils were utilized as actuation elements. Incorporating SMA into host materials including composites has so far been a challenge to the poor bonding characteristics of SMA [15]. In this work, flexible SMA strips were prepared and embedded together with the sensor strips in polydimethylsiloxane (PDMS), as illustrated in Figure 6, to create robotic fingers that could in future be used to develop prosthetic hands where the amount of bending can be controlled by adjusting the electrical current flowing through the SMA. NiTi sheets from Memetis GmbH (Karlsruhe, Germany) with 100 μm thickness (±15 μm) were used as base material for this study. Transformation temperatures are crucial for SMA materials. They define the start and finish of transformation between the martensite and austenite phases during heating and cooling, and their inherent hysteresis is significant for SMA applications. To enable self-fixation and ensure the stability of the sensor, the SMA metal strips were integrated within a two-layer PDMS encapsulation. The process involved placing the SMA under the sensor strip, which was then encapsulated with 200 µm PDMS during assembly. After the PDMS was cured, the assembly was cut using the femtosecond laser and released from the glass wafer.

## 3. Results and Discussion

### 3.1. Single Sensor Strip Characterization

**Figure 7:**
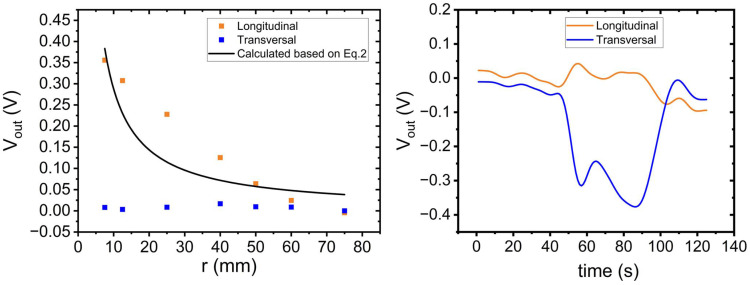
(**Left**): $V_{\mathrm{out}}$ obtained at certain bending radii r for strain gauges oriented in longitudinal (orange) and transversal (blue) orientation. For comparison $V_{\mathrm{out}}\left(r\right)$ as obtained from (Equation (2)) assuming A = 100 and GF = 2.5 is also given, (**Right**): The course of ${\;V}_{\mathrm{out}}$ as obtained during a twisting experiment with angles varied from 0° to 270° and back to 0°.

Single sensor strips were first evaluated using test tubes of different radii. The sensor strips were manually wrapped around tubes and fixed using PDMS without requiring any external force to maintain the bent shape. Figure 7 (left) shows the measurement results for the sensor strips as they were bent over the test tubes after compensating an offset that was obtained in a flat position. The decrease in output voltage with the increasing bending radius is consistent with Equation (2) assuming an amplification factor of A = 100, an interlayer thickness of h = 11.5 μm, a gauge factor of GF = 2.5, and a supply voltage of V_ex_ = 2.5 V. Additionally, the figure shows that transversal sensor nodes show no change in the output signal. Sensor strips were also twisted to examine whether there is a significant difference in the output voltages of differently oriented nodes. The sensor strip was clamped at both ends to induce a twist. The longitudinal nodes exhibited lower output voltage during twisting because they experienced minimal deformation (A positive output voltage indicates tension in the outer region and compression in the inner region of the twisted material), while transversal nodes went through a stronger bending deformation. The output voltage of the longitudinal node exhibited little to no change, while the voltage of the transversal node approached −400 mV. As the sensor was slowly twisted from 0° to 270° and back to 0°, the absolute value of the voltage gradually increased with the twisting angle (see Figure 7 (right)).

The obtained output voltage readings showed good reproducibility across multiple trials. In previous research it had already been shown that sensors based on this technology exhibit negligible hysteresis and no delays in dynamic tests at relevant deformation speeds [19].

### 3.2. Smart Finger Shape Feedback Sensor

**Figure 8:**
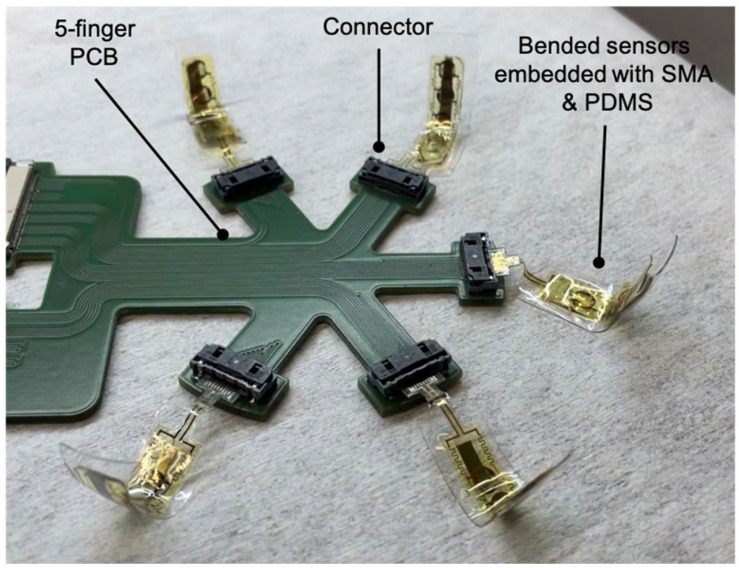
Five sensors connected to the five-finger PCB.

The SMA strip possesses the unique property of bending into various shapes and maintaining its form, providing deformation situations recognized by signals from the sensor strip. SMA strips were used as a template to bend the fingers. In this way, this represented only a first step to develop prosthetic hands or robotic manipulators to replicate the natural bending motion of human fingers (see Figure 8).

**Figure 9:**
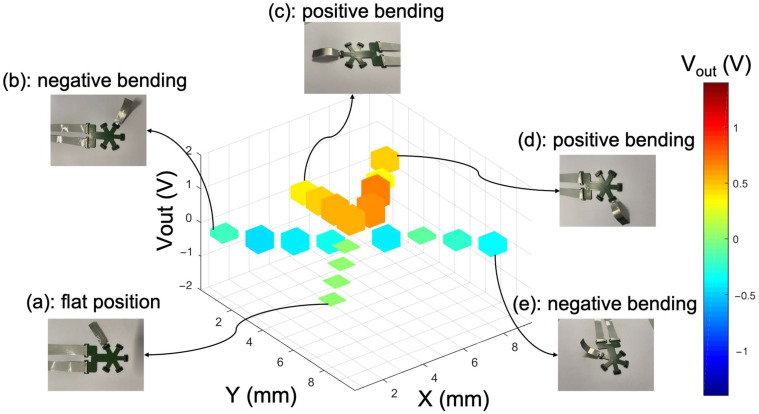
Sensor signals for every sensor strip with the use of SMA in different scenarios: flat position (**a**), positive bending (**c**,**d**), or negative bending (**b**,**e**). The color coding of the columns corresponds to the output voltage. The red color indicates positive bending, while the blue color indicates negative bending.

The sensor’s measurement range extended from −1.25 V to 1.25 V, in which bending radii could be precisely determined. The derivation of bending radii was based on parameter values, including an amplification factor of A = 100, interlayer thickness of h = 11.5 μm, gauge factor of GF = 2.5, and a supply voltage which was set to a value of V_ex_ = 2.5 V. The sensor strips and flexible PCB, which consisted of 20 individual nodes for detecting spatial tactile information, were tested. Figure 9 shows five different images that capture various states of deformation. The first image serves as the baseline reference with an offset of ±0.1 V corresponded to 8% of the output voltage, showing the sensor in a flat position. The second and fifth images exhibit negative bending, while the third and fourth images represent positive bending configurations. These fixed bending variations are induced by activating the SMA material component in a controlled manner, demonstrating the sensor’s ability to undergo dynamic and reversible deformations. To activate the SMA the structure was exposed to 100 °C for a very short time. The temperature exposure did not affect the sensor performance. The thin PDMS layer provides practically no restoring force to the fingers. The raw signals obtained from the piezoresistive sensor are presented in 3D graphical form, providing insights into the sensor’s response to mechanical stimuli obtained in real time.

The smart finger’s length and width were 41 mm and 7 mm respectively, while its mass was 22 mg (and 230 mg with SMA) and a minimum bending radius of curvature without causing any damage of 10 mm allowed for flexibility, making it a versatile smart device for different applications. The palm, without the five fingers, had a length of 92 mm, a width of 65 mm, and a weight of 3.7 g. With a total length of 64 mm, the smart fingers can mimic gripping by a human hand.

### 3.3. Object Sensing in a Flexible Granular Material Gripper

**Figure 10:**
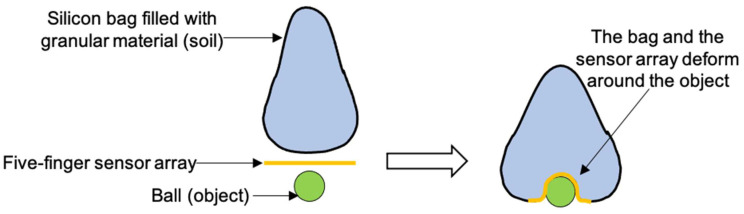
The working principle of the sensing system in combination with a granular pressure bag for object conformation.

Robotic gripping often imitates the human hand’s ability to grasp objects of various shapes and sizes. This property has already been proven for a gripper with a flexible membrane or a bag filled with granular material such as sand or coffee grounds [20]. With changes in air pressure, the granular material can change between a fluid-like to a rigid solid state. When the gripper is relaxed (low air pressure), it can gently conform to the object’s shape and while increasing the air pressure, the granular material jams together creating a solid and rigid structure that firmly holds the object in place. In this study, the smart finger system was placed beneath a bag filled with granular material. A smart finger system placed between the gripper and the object adapts to the shape of the object. as sketched in Figure 10. Thereby, the sensor feedback created could in future be useful when the measurement and control of contact is required, such as in robotics for medical applications or industrial automation. A mathematical model was used to reconstruct surface shape using local sensor data. First, from the output voltages of two sensor nodes the local radii of curvature were determined with the help of Equation (2).

For the second step, a the third-order polynomial function was assumed to represent the longitudinal profile f(x) of the finger strip as

(3) $$f\left(x\right)=a_{3}x^{3}+a_{2}x^{2}+a_{1}x+a_{0}$$

Since $a_{o}$ (reflecting offset) and $a_{1}$ (reflecting tilt) are not influenced by bending and not relevant for the shape, they can be set to zero. Two bending radii measurements were taken at different positions along the strip, $r_{1}$ measured at x = 0 and $r_{2}$ measured at x = 1. Assuming that the bending radius at position x can be approximated by ${\left[\ddot{f\left(x\right)}\right]}^{-1}$ the remaining parameters $a_{2}\;$ and $a_{3}$ in Equation (3) are given as

(4) $$a_{2}=\frac{1}{2r_{1}}\;,\;a_{3}=\frac{1}{6}\left(\frac{1}{r_{2}}-\frac{1}{r_{1}}\right)$$

**Figure 11:**
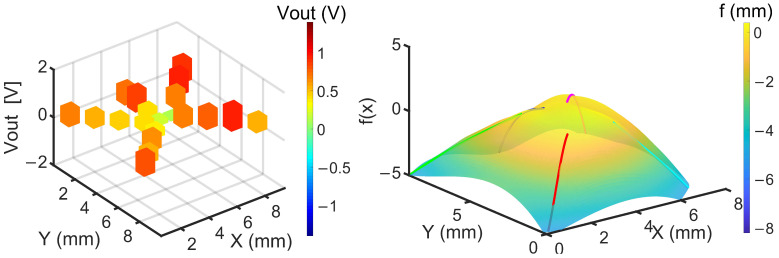
(**Left**): Raw sensor signals. (**Right**): Surface reconstruction based on an interpolation between the five linear shape profiles representing the shape of the gripped hemisphere with the color bar indicating the position in the *f* direction.

In the initial state (before contact with the spherical object), the output voltages of the sensor strips were close to zero. Figure 11 shows sensor signals obtained from five fingers and the longitudinal strip shapes determined by them according to Equations (2)–(4). Note that the *x*-direction in Equation (3) is always the long axis of the sensor strip, whereas in Figure 11 the long axes of the sensor strips are rotated within a fixed *x*/*y* coordinate system. Together, the sensor data were able to reproduce the shape of the gripped hemisphere with good fidelity, demonstrating the capability and effectiveness of the artificial hand with piezoresistive sensor fingers.

### 3.4. 3D Mapping of an S-Shaped Surface

**Figure 12:**
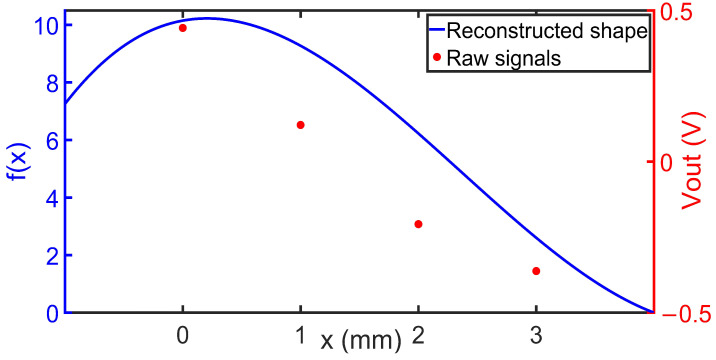
Red: Raw sensor signals S_n1_ (n = 1, …, 4) Blue: reconstructed strip shape as it is fixed to an S-shaped body. Only signals from S_11_ and S_13_ which are aligned in longitudinal direction with the finger strip were used for the reconstruction.

For demonstrating another capability of the developed sensor fingers, a single strip was placed over an S-shaped surface. Bending radii measurements were taken at two positions along the strip, Sn1 & Sn3. Figure 12 shows the shape that was reconstructed from the two sensor signals as described above.

Figure 12 indicates that the sensor nodes S_11_ sand S_21_ showed positive bending values, while signals of S_31_ and S_41_ indicated negative bending. Signals from S_12_ and S_14_ were weaker because these sensor nodes are aligned in the vertical direction with the finger strip. The reconstructed surface closely matched the shape of the bending applied to it.

## 4. Conclusion

This paper has introduced a new hybrid system that showcases the remarkable potential of self-sensing flexible piezoresistive sensor strips. These strips, made of thin PI foil, can mimic the intricate behavior of artificial skin on a robotic hand gripper. Because of the flexible nature of the PI foil, which is only about 17 µm thick, it is highly suitable for integration onto human skin, offering numerous advantages in terms of mechanical performance and versatility. Our research combined microfabrication techniques in a controlled cleanroom environment with integration and packaging methods, such as those provided by PDMS embedding and multilayer PCB, to create these innovative devices. The presence of sensor nodes, each using a full Wheatstone bridge configuration, provides a significant advantage in terms of signal strength and temperature compensation. This opens up promising application possibilities, especially in conjunction with thermally excited actuation in soft robotic hand grippers. Such systems require precise feedback so that gripping movements can be accurately controlled. In addition, we introduced a pioneering method for reconstructing the sensor array’s surface. By leveraging signals from multiple integrated bending sensors and fundamental geometric principles, the results of our surface reconstruction align closely with the applied bending shapes, ensuring robust surface reconstruction even in cases where individual sensors may not be practical or may even malfunction. Still, this is just one very basic example of the surface reconstruction algorithm; other methods, including AI, may also be used in the future. Furthermore, our study explored the integration of Shape Memory Alloy (SMA) foils alongside our sensors to mimic human hand movements. This involved controlling the sensor strips through temperature manipulation. By embedding them within Polydimethylsiloxane (PDMS), we enhanced the sensor’s tactile feel and adhesive properties, utilizing the shape memory effect of SMA primarily through heating processes. This innovative approach opens up new possibilities including more accurate surface reconstruction, enhanced reliability, and the ability to mimic human hand gripping motions, enabling us to create hand-like devices capable of interacting with various objects. These findings are promising for future applications, particularly in robotics and prosthetics. The ability to accurately mimic human hand movements and reconstruct sensor surfaces paves the way for robotic hand functionality.

## Data Availability

The raw data of the experiments can be requested from the authors.
